# VDAC1 oligomerization may enhance DDP‐induced hepatocyte apoptosis by exacerbating oxidative stress and mitochondrial DNA damage

**DOI:** 10.1002/2211-5463.13359

**Published:** 2022-01-14

**Authors:** Xueqin Zhu, Lei Luo, Yanyan Xiong, Nan Jiang, Yurun Wang, Yuan Lv, Ying Xie

**Affiliations:** ^1^ Key Laboratory of Molecular Epidemiology of Hunan Province School of Medicine Hunan Normal University Changsha China; ^2^ Changsha Center for Disease Control and Prevention Beijing China

**Keywords:** 4,4'‐diisothiocyanatostilbene‐2,2'‐disulfonic acid, apoptosis, cisplatin, hepatocyte, oligomerization, voltage‐dependent anion channel 1

## Abstract

Cisplatin (DDP)‐based chemotherapy is a preferred treatment for a broad spectrum of cancers, but the precise mechanisms of its hepatotoxicity are not yet clear. Recently, the role of voltage‐dependent anion channel protein 1 (VDAC1) in mitochondrial activity and cell apoptosis has attracted much attention. Our aim was to investigate the effects of mitochondrial outer membrane protein VDAC1 oligomerization in DDP‐induced hepatocyte apoptosis. L‐02 hepatocytes were divided into 4 groups: (a) control group, (b) 4,4'diisothiocyanate‐2,2'‐disulfonic acid (DIDS; 40 μm) group, (c) DDP (5 μm) group, and (d) DDP and DIDS combination group. Cell apoptosis was tested by Annexin V/FITC assay, protein expression of caspase‐3, γH2AX and NDUFB6 were observed by western blot assay, reactive oxygen species (ROS), and mitochondrial superoxide anion radical (O_2_^•−^) were detected by DCFH‐DA and MitoSOX probe, and DNA damage was assessed by comet assay. Moreover, the activity of mitochondrial respiratory chain complex I was determined by the colorimetry method. Compared with the control group, apoptosis rate and activated cleaved‐caspase‐3 protein, ROS and O_2_^•−^ generation, DNA damage marker comet tail length, and γH2AX protein level increased in the DDP treatment group (*P* < 0.05). Activity of mitochondrial COXI decreased after DDP treatment (*P* < 0.05). DIDS, as a VDAC1 oligomerization inhibitor, antagonized DDP‐induced apoptosis by diminishing oxidative stress and DNA damage and protecting mitochondrial complex protein. These results show that VDAC1 oligomerization may play an important role in DDP‐induced hepatocyte apoptosis by increasing ROS and mtDNA leakage from VDAC1 pores, exacerbating oxidative stress and mtDNA damage.

## Graphical abstract

This study found that DDP might convert VDAC1 monomers to VDAC1 oligomers, which promoted ROS and mtDNA fragments to be released from the mitochondria to the cytoplasm through VDAC1 channel, resulting in the expansion of mitochondrial oxidative stress and DNA damage. DIDS, an inhibitor of VDAC1 oligomerization, can alleviate DDP‐induced mitochondrial oxidative stress, DNA damage, and cell apoptosis in L‐02 hepatocytes.

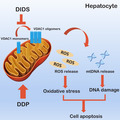

**Abbreviations**
COXI: complex I
DDP: cisplatin
DIDS: 4,4' ‐diisothiocyanate‐2,2' ‐disulfonic acid
ECL: electrochemiluminescence
NAD+: nicotinamide adenine dinucleotide
NADH: nicotinamide adenine denucleotide reduced form
O_2_^•^^−^
: mitochondrial superoxide anion radical
OD: optical density
pH: potential of hydrogen
PI: propidium iodide
ROS: reactive oxygen species
RPMI: Roswell Park Memorial Institute
RT: room temperature
VDAC1: voltage‐dependent anion channel protein 1

With the development of medical treatments, cisplatin (DDP)‐based chemotherapy is a preferred treatment for a broad spectrum of cancers. Unfortunately, the clinical use of DDP is limited by its severe toxic side effects. Liver and kidney toxicities are the most common side effects and dose‐limiting factor under DDP treatment and reduced therapeutic effect and life expectance of patients. However, the exact mechanism of how to protect against liver toxicity has not been clarified clearly [1].

Generally, the interactions with purine residues to form DNA cross‐linking complexes are considered as the major biological mechanism of DDP [2]. Mitochondria are the central for energy supply and signal transduction in hepatocytes, which also serves as targets for chemotherapeutic drug. If DNA damage repair fails, the mitochondrial‐dependent apoptotic process is initiated [3]. When DNA damage and subsequent mitochondrial oxidative stress occurs, electron leakage from the respiratory chain gradually derives into superoxide anion free radical (O_2_^•−^), hydroxyl radical, and hydrogen peroxide. Reactive oxygen species (ROS) further interact with DNA, lipids, and proteins and cause a cascade of mitochondrial damage and release of apoptotic factors, leading to mitochondria‐dependent apoptosis [4]. Numerous studies have confirmed that antioxidants reduced DDP‐induced hepatotoxicity by inhibiting oxidative stress, reducing inflammatory response and apoptosis [5, 6, 7].

Recently, the role of voltage‐dependent anion channel protein 1 (VDAC1) in mitochondrial activity and cell apoptosis has attracted much attention. VDAC1 is an important channel for exchange of energy and substances between mitochondria and cells by regulates transport of anions, cations, ATP, and other metabolites [8]. Various apoptosis inducers, including staurosporine, curcumin, selenite, and arsenic oxide, induced formation of the VDAC dimer and oligomers [9]. Keinan N et al. also found that DDP induced VDAC1 oligomerization in HEK‐293 cells at relatively high concentrations (40–50 μm) [10]. VDAC1 oligomer forms a pore large enough for the release of apoptosis‐inducing factors, which activates the caspase cascade reaction and subsequently leads to apoptosis [11].

The well‐known chloride channel blocker 4,4'‐diisothiocyanatostilbene ‐2,2'‐disulfonicacid (DIDS) was used to investigate the role of VDAC1 oligomerization in cytotoxicity. DIDS affects the activity of a number of transporters, and there is an extensive literature in support that DIDS can inhibit VDAC1 oligomerization. DIDS reversibly interacts with VDAC1 and inhibits its channel conductance and oligomerization [12]. Ben‐Hail et al. demonstrated that VDAC1 undergoes oligomerization in response to cancer cell damage and mitochondrial oxidative stress, and DIDS and its analogs inhibited apoptosis via direct interaction with VDAC1 to inhibit its oligomerization and subsequent Cyto c release and apoptosis [11]. However, the role of VDAC1 oligomerization in hepatoxicity exposed after DDP has not been illustrated.

The present study aims to further explore the potential effects of VDAC1 oligomerization on DDP‐induced mitochondrial damage and apoptosis in L‐02 hepatocytes, which provide scientific evidence for alleviating liver injury in patients undergoing chemotherapy.

## Materials and methods

### Cell culture and treatment

L‐02 cells were grown in RPMI 1640 medium supplemented with 10% fetal bovine serum and 1% penicillin/streptomycin. DDP (Merck, USA) and DIDS (MedChem Express, USA) were used in the experiments.

### Cell apoptosis analysis

Cell apoptosis was detected by Annexin V‐FITC/PI Apoptosis kit (Beijing Solarbio Technology Co., Ltd., China). In brief, 1 × 10^5^ cells were resuspended in 100 μL supplied binding buffer and then stained with 5 μL FITC‐conjugated Annexin V and 10 μL PI at RT for 15 min in darkness, according to the manufacturer’s protocol. The fluorescence intensities of the cells were detected by flow cytometer (ACEA Biosciences, USA). The Annexin V‐FITC^‐^/PI^‐^ cell population was regarded as normal, while Annexin V‐FITC^+^/PI^‐^ cells were taken as a measure of early apoptosis, Annexin V‐FITC^+^/PI^+^ as late apoptosis, and Annexin V‐FITC^‐^/PI^+^ as necrosis.

### Western blotting

Methods for Western blotting were performed as described previously [13]. All antibodies were purchased commercially: anti‐Caspase‐3 (Santacruz; sc‐56053); anti‐γH2AX (Merck Millipore; 2391108); anti‐NDUFB6 (Abcam, ab110244); anti‐β‐actin (GeneTex; GTX124502). After incubation with the secondary antibody, the protein bands were visualized with ECL detection (Invitrogen, USA) and a chemiluminescence imager (Minichem, China).

### Measurement of intracellular ROS levels

The intracellular ROS levels were measured using a Reactive Oxygen Species Assay Kit (Beyotime Biotechnology, China) as described previously [13]. Following the treatment, cells were incubated with DCFH‐DA for 20 min at 37 °C. Fluorescence was analyzed using a multifunctional enzyme labeling instrument (Molecular Devices, USA) with excitation at 488 nm and emission at 525 nm. The fluorescence intensity reflected the amount of ROS generated.

### Detection of MitoSOX formation

Mitochondrial superoxide anion radicals (MitoSOX) were detected using MitoSOX™ Red mitochondrial superoxide indicator (Thermo Scientific, USA) as described previously [13]. 5 mm MitoSOX working solution was added to cells and incubated at 37 °C for 10 min, protected from light. After washing, cells were detected with a multifunctional molecular device with excitation at 510 nm and emission at 580 nm.

### Comet assay

The comet assay was performed using a reagent kit for single‐cell gel electrophoresis (Trevigen, Inc., Gathersburg, MD, USA) according to a modified method of Viera et al. [14]. Cells were trypsinized and collected, washed with PBS, and resuspended on PBS. Cells were added to melted LMAgarose (Trevigen) cooled to 37 °C at a ratio of 1 : 10 and pipetted onto a pre‐warmed comet slide and spread evenly. Slides were then placed at 4 °C for 30 min to allow adherence of the agarose to the slides. The slides were then gently immersed in lysis solution overnight at 4 °C. Following lysis, the slides were immersed in 1× Neutral Electrophoresis Buffer containing Tris base and sodium acetate (corrected to pH 9 with glacial acetic acid) for 30 min at 4 °C. The slides were electrophoresed at 21 V for 45 min at 4 °C in the neutral electrophoresis buffer and then immersed in DNA precipitation solution and 70% ethanol successively for 30 min each time. Then, slides were stained with DAPI and viewed using fluorescent microscopes. Comet measurement and quantitative analysis were performed using casp software.

### Complex I enzyme activity assay

Mitochondrial OXPHOS Complex I enzyme activity was determined using the Complex I Enzyme Activity Microplate Assay Kit (Abcam109721; UK). The enzymatic activity was expressed as the change in absorbance (mOD) per minute, which was calculated by fitting the initial linear portion of each kinetic curve.

### Statistical analysis

The results were calculated from quantitative data obtained from three replicate experiments. Statistical analysis was performed using ANOVA and LSD *t*‐test in SPSS v20.0 software. The *P*‐values ≤ 0.05 were considered significant.

## Results

### DIDS antagonized DDP‐induced apoptosis in L‐02 hepatocytes

**Fig. 1:**
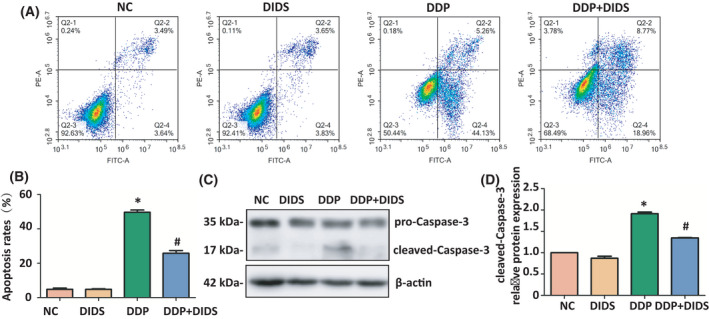
DIDS antagonized cisplatin‐induced apoptosis in L‐02 hepatocytes. (A‐B) The rate of apoptosis was measured by Annexin V‐FITC/PI staining in L‐02 hepatocytes exposed to 40 μm DIDS group and/or 5 μm DDP group for 72 h. Comparisons were made using ANOVA with LSD *t*‐test. Error bars indicate standard deviation (SD), *n* = 6. (C) Western blotting analysis for apoptotic proteins (pro‐caspase‐3 and cleaved caspase‐3) in L‐02 hepatocytes. (D) Quantification of cleaved‐caspase‐3 relative protein expression. *P < 0.05 versus the NC group and #*P* < 0.05 versus the DDP group. ANOVA with LSD. Error bars indicate SD, *n* = 3.

We treated L‐02 hepatocytes with 40 μm DIDS and/or 5 μm DDP for 72 h to investigate the effects of DIDS on DDP‐induced hepatocyte apoptosis. With Annexin V‐FITC/PI detection, the rate of apoptosis significantly increased in the DDP group compared with the control group; however, cells treated with the combination of DDP and DIDS displayed significant decreased rates of apoptosis compared with the DDP group (*P* < 0.05, Fig. 1A,B). Additionally, the protein expression levels of cleaved caspase‐3, a marker of apoptosis, significantly decreased in DDP‐ and DIDS‐treated group compared with the DDP group (*P* < 0.05, Fig. 1C,D). These results presented that DIDS antagonized DDP‐induced apoptosis in L‐02 hepatocytes.

### DIDS inhibited DDP‐induced mitochondrial oxidative stress in L‐02 hepatocytes

**Fig. 2:**
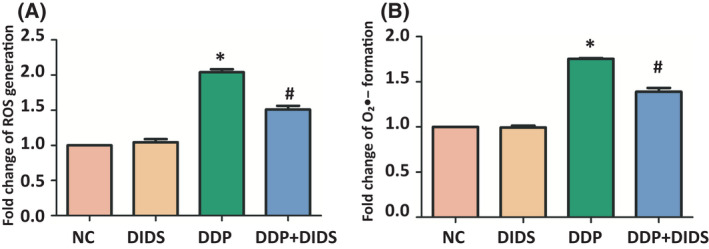
DIDS inhibited cisplatin‐induced mitochondrial oxidative stress in L‐02 hepatocytes. (A‐B) ROS and O_2_^•−^ generation was detected using DCFH‐DA probe and MitoSOX Red probe, respectively, in L‐02 hepatocytes exposed to 40 μm DIDS group and/or 5 μm DDP group for 72 h. *P < 0.05 versus the NC group and ^#^ *P* < 0.05 versus the DDP group. ANOVA with LSD. Error bars indicate SD, *n* = 6.

The mitochondrial electron transport chain is the major intracellular source of ROS, including O2^•−^, hydroxyl radical, and various peroxides and hydroperoxides. To assess the mitochondrial oxidative stress in L‐02 hepatocytes, ROS and O2^•−^ generation were detected using the DCFH‐DA probe and the MitoSOX Red probe, respectively. ROS and O_2_^•−^ generation increased significantly in the DDP group, compared with the control group. However, ROS and O_2_^•−^ generation significantly decreased in DDP‐ and DIDS‐treated group compared with the DDP group (*P* < 0.05, Fig. 2A,B). These results presented that DIDS inhibited DDP‐induced mitochondrial oxidative stress in L‐02 hepatocytes.

### DIDS alleviated DDP‐induced DNA damage in L‐02 hepatocytes

**Fig. 3:**
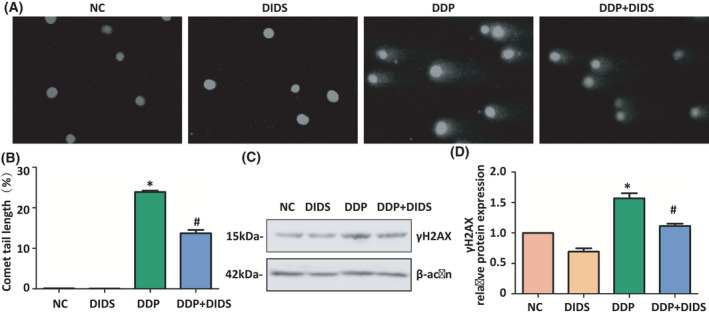
DIDS alleviated cisplatin‐induced DNA damage in L‐02 hepatocytes. (A‐B) The tails length of comet was measured by comet assay in L‐02 hepatocytes exposed to 40 μm DIDS group and/or 5 μm DDP group for 72 h. ANOVA with LSD. Error bars indicate SD, *n* = 6. (C) Western blotting analysis of γH2AX protein in L‐02 hepatocytes. (D) γH2AX relative protein expression. *P < 0.05 versus the NC group and *^#^P* < 0.05 versus the DDP group. ANOVA with LSD. Error bars indicate SD, *n* = 3.

DNA double‐strand breaks were measured using the neutral comet assay. The larger the comet tail, the more DNA damage has occurred. The comet tail length significantly increased in the DDP group compared with the control group; however, the group treated by DDP and DIDS in combination displayed significant decreased comet tail length compared with the DDP group (*P* < 0.05, Fig. 3A,B). Additionally, the protein expression levels of γH2AX, as an early marker of the DNA damage response, significantly increased in the DDP group compared with the control group; however, the expression of γH2AX level significantly decreased in DDP‐ and DIDS‐treated group compared with the DDP group (*P* < 0.05, Fig. 3C,D). These results presented that DIDS alleviated DDP‐induced DNA damage in L‐02 hepatocytes.

### DIDS rescued DDP‐induced impaired mitochondrial respiration chain in L‐02 hepatocytes

**Fig. 4:**
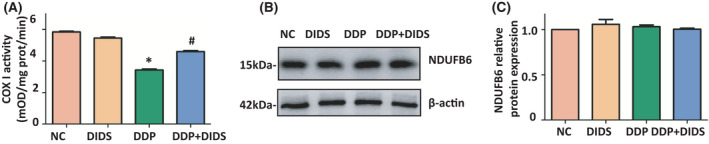
DIDS reduces cisplatin‐induced impaired mitochondrial respiration chain in L‐02 hepatocytes. (A) Mitochondrial respiratory chain COXI activity was detected. *P < 0.05 versus the NC group and ^#^ *P* < 0.05 versus the DDP group. ANOVA with LSD. Error bars indicate SD, *n* = 6. (B) Western blotting analysis of COXI subunit NDUFB6 protein in L‐02 hepatocytes. (C) Quantification of NDUFB6 relative protein expression. ANOVA with LSD. Error bars indicate SD, *n* = 3.

COXI is the largest mitochondrial respiratory chain complex and is the entry point for electrons into the respiration chain. COXI activity was determined by following the oxidation of NADH to NAD^+^ and the simultaneous reduction of the provided dye which leads to increased absorbance at OD 450 nm. We found that COXI activity was significantly suppressed in DDP‐treated cells compared with NC cells, while DIDS antagonized the effects of DDP (*P* < 0.05, Fig. 4).

Considering the enzyme activity was also influenced by enzyme content, we also detected the protein level of representative COXI subunit NDUFB protein [15]. Western blot analysis showed no significant difference in NDUFB6 protein level among different treatment groups, which indicated that DIDS rescued the decrease of mitochondria respiratory activity under DDP exposure.

## Discussion

In this study, DIDS, as an inhibitor of VDAC1 oligomerization, reduced the DDP‐induced increase of O_2_^•−^ release and ROS generation and alleviated comet tail length and increase of γH2AX protein. DNA damage and mitochondrial respiratory chain COXI activity protection by DIDS, gave rise to inhibition of DDP‐induced activation of caspase 3 and hepatocytes apoptosis.

VDAC1 oligomerization inhibitors DIDS inhibited DDP‐induced apoptosis in L‐02 hepatocytes, which indicated VDAC1 oligomerization might be a major target in DDP‐induced hepatocytes apoptosis. In previous studies, DIDS has been shown to antagonize apoptosis. Liu AH et al. demonstrated that DIDS attenuated staurosporine‐induced cardiomyocyte apoptosis by PI3K/Akt signaling pathway [16]. DIDS inhibits overexpression BAK1‐induced mitochondrial apoptosis through GSK3β/β‐catenin signaling pathway [17].

In the present study, we focused on the role of DIDS in DDP‐induced mitochondrial damage and apoptosis. Mitochondria are the regulatory centers of cellular energy metabolism and mainly targeted to induced apoptosis [18]. Functional impairment of mitochondria due to inhibition of respiratory chain complexes increases the electrons leakage from electron transport chain and promotes the production of ROS, causing oxidative stress [19]. DIDS antagonized quinocetone induces apoptosis in HepG2 cells by alleviated cellular oxidative stress [20]. VDAC1 is located in the outer membrane of mitochondria and constitutes a protein channel for the transport of metabolites between the mitochondria and cytoplasm [21]. It was found that VDAC1 oligomerization promoted ROS to be released from the mitochondria to the cytoplasm through VDAC1 channel, resulting in the expansion of oxidative damage cascade [22]. Feng et al. found that iron death inhibitors protected cardiomyocytes by reducing VDAC1 oligomerization and mitochondrial ROS generation [23]. We also found that intervention of VDAC1 oligomerization by DIDS significantly decreased ROS and O_2_^•−^ release after DDP treatment, suggesting that VDAC1 oligomerization might promote oxidative damage by increasing electron leakage out from mitochondria.

Moreover, DDP can directly attack DNA to form DNA adduct, while mitochondrial DNA encodes 13 proteins that are essential subunits of mitochondrial respiratory chain complexes. DNA oxidative damage of mitochondrial further leads to dysfunction of mitochondrial respiratory chain complex, affecting electron transport and energy production [24]. In this study, DIDS alleviated DDP‐induced DNA damage and COXI activity decreased in hepatocytes, suggesting that VDAC1 oligomerization might promote DDP‐induced mtDNA damage and subsequently mitochondrial respiratory chain complex impairment. DIDS alleviated DNA damage induced by T‐2 toxin in human gastric epithelium GES‐1 cells by inhibiting VDAC1 oligomerization [25]. Kim et al. also found that damaged mtDNA broke into fragments, which stimulated VDAC1 oligomerization form mitochondrial pores to release mtDNA fragments into the cytoplasm, thus aggravating mitochondrial damage [26]. It is reasonable to deduce that DDP‐induced VDAC1 oligomerization promoted mtDNA leakage out of mitochondria, which aggravated mtDNA damage. Meanwhile, mtDNA leakage after DDP exposure affected respiratory chain complex activity encoded by mtDNA and further promoted electrons leakage and mitochondrial oxidative damage.

In conclusion, this study found that DDP might elevate VDAC1 oligomerization, which promoted ROS and mtDNA to be released from the mitochondria to the cytoplasm through VDAC1 channel, resulting in the expansion of mitochondrial oxidative stress and DNA damage. DIDS, an inhibitor of VDAC1 oligomerization, can alleviate DDP‐induced mitochondrial oxidative stress, DNA damage, and cell apoptosis in L‐02 hepatocytes, which provide scientific evidence for alleviating liver injury in patients undergoing chemotherapy.

## Conflict of interest

The authors declare no conflict of interest.

## Authors' contributions

LL conceived the idea of the study. XQZ analyzed most of the data and wrote the initial draft of the paper. YX and YL performed the research and reviewed the manuscript. The remaining authors contributed to the collection of data. All authors read and approved the final manuscript.

## Data Availability

The raw data supporting the conclusions of this article will be made available by the authors, without undue reservation.
